# Identification of E2F transcription factor 7 as a novel potential biomarker for oral squamous cell carcinoma

**DOI:** 10.1186/s13005-021-00258-2

**Published:** 2021-02-26

**Authors:** Ping Zhou, Lei Xiao, Xiaonan Xu

**Affiliations:** 1 Department of Stomatology, Jining No.1 People’s Hospital, Jining, 272000 Shandong China; 2 Department of Stomatology, Yantai Yuhuangding Hospital, Yantai, 264000 Shandong China

**Keywords:** Oral squamous cell carcinoma, E2F transcription factor 7, Growth, Invasion, Migration, Epithelial–mesenchymal transition

## Abstract

**Background:**

As a tumor-accelerating transcriptional factor, E2F transcription factor 7 (E2F7) was up-regulated in many forms of cancers. Nevertheless, little has been reported about the impacts of E2F7 on oral squamous cell carcinoma (OSCC). Here, we aimed to probe whether E2F7 had influences on OSCC and its potential mechanism.

**Methods:**

The expression of E2F7 in OSCC tissues was analyzed using the data acquired from TCGA and ONCOMINE databases. E2F7 prognostic value in OSCC patients was analyzed utilizing TCGA database. The expression of E2F7 in OSCC cell lines was detected by qRT-PCR. Gain-and loss-function of E2F7 assays in TCA-83 and CAL27 cells were performed respectively to inquire the function of E2F7. Western blotting was applied to test the alternations of EMT-related markers.

**Results:**

In OSCC tissues, E2F7 was highly expressed. Besides, high expression of E2F7 predicted worse prognosis in OSCC patients. Moreover, E2F7 was over-expressed in TCA-83, HSC-4 and CAL27 (all OSCC cell lines) cells relative to that in HNOK (a normal cell line) cells. Gain-and loss-function assays displayed that deficiency of E2F7 suppresses CAL27 cell growth, migration, invasion and E2F7 high-expression resulted in inverse outcomes in TCA-83 cells. Finally, we found that silencing of E2F7 facilitated E-cadherin protein expression level and reduced N-cadherin, Vimentin and Snail protein levels in CAL27 cells, whilst E2F7 high-expression exhibited the opposite effects in TCA-83 cells.

**Conclusions:**

These outcomes indicated that E2F7 performs a carcinogenic role in OSCC, which provides a theoretical basis for the therapeutic strategies of OSCC.

## Introduction

Oral squamous cell carcinoma (OSCC) represents as one of the most prevalent cause of cancer death around the world, especially in males (M:F = 10.8:1) [1, 2]. There were 177,384 new deaths discovered globally in 2018 [3]. Although there are many improved treatments in clinical practice (such as surgery, chemotherapy and radiotherapy), multidisciplinary collaboration and sequential therapy can ameliorate the prognosis, the 5 year survival rate of OSCC is merely 50% [4, 5]. Furthermore, after treatment, the probability of local recurrence and distant metastasis in patients is as high as 25–50% [6]. Due to the lack of early molecular markers, most OSCC cases are detected at late stage, further increasing the probability of death. In improving patient survival, early detection is very effective. Hence, it is important to screen more effective biomarkers to ameliorate the detection efficiency for OSCC patients.

The E2F transcription factor family is vital for the modulation of cell growth, differentiation, apoptosis and DNA damage responses [7]. E2F transcription factors exerted effects in regulating cell angiogenesis, apoptosis and cell cycle in cancers [8]. As the study reported by Zhi et al. [9], E2F3 was highly expressed in head and neck squamous cell carcinoma. Given the momentous effects of E2F2 in cancers, Li et al. [10] suggested that E2F2 polymorphisms could be utilized to predict the risk of oropharynx squamous cell carcinoma recurrence. There is one literature has reported that certain E2Fs might have influences on OSCC [11]. Kaplan-Meier analysis showed that E2F gene set high expression were concerned with worse prognosis in OSCC patients [11]. Nevertheless, there are few reports about the impacts of E2Fs on OSCC, and the related mechanism has not been clarified, and needs further elaboration.

As one member of E2F family, E2F transcription factor 7 (E2F7) is significant for managing cell proliferation, differentiation and cell cycle progression [12]. Among many types of malignancies, E2F7 is frequently up-regulated, thus it is a tumor-promoting transcription factor [13, 14]. A recent study manifested that E2F7 expression level was significantly fortified in thyroid cancer cells [15]. It has demontrated that E2F7 was involved in the development of cervical cancer [16]. However, the influences of E2F7 on OSCC were rarely elucidated.

The present paper discovered that E2F7 was over-expressed in OSCC tissues and cells. Moreover, E2F7 depletion retrained CAL27 cell growth, migration and invasion whilst E2F7 high-expression was revealed to accelerate TCA-83 cell growth, migration and invasion, which were realized by regulating epithelial–mesenchymal transition (EMT). Most importantly, this article first reports the influences of E2F7 on OSCC. Together, our findings offer prospects on that E2F7 may act as an novel marker for OSCC carcinogenesis.

## Methods

### Specimens

TCGA database (https://cancergenome.nih.gov/) is applied to analyze the expression levels of E2F7 in 340 OSCC tissues and 32 normal specimens. Subsequently, employing the data from ONCOMINE (https://www.oncomine.org), we analyzed E2F7 levels in OSCC tissues (*n* = 57) and normal specimens (*n* = 22). The overall survival (OS) of OSCC tissues was analyzed by Kaplan-Meier. Differences between groups were evaluated by utilizing Log-rank test. Basing on the median value of E2F7 expression, OSCC patients were divided into high expression group (*n* = 31) and low expression group (*n* = 32).

### Cell lines

OSCC cell lines TCA-83, HSC-4, CAL27 and the human normal oral keratinocytes (HNOK) cell line were gained from the Chinese Academy of Sciences (Shanghai, China). Then the cells were cultivated in RPMI-1640 medium (Gibco, NY, USA), which including 10% FBS, 100 U/ml penicillin and 0.1 mg/ml streptomycin (Gibco, NY, USA) under normal condition.

### Cell transfection

si-E2F7#1 (5′-CCTCTATGACATAGCCA-3′), si-E2F7#2 (5′-CATCTATGACATTGTAA-3′), si-con (5′-CGAACUCACUGGUCUGACC-3′), pcDNA3.1-E2F7 and pcDNA3.1 were acquired from GenePharma Co., Ltd. (Shanghai, China). Then by employing lipofectamine 3000 (Invitrogen, USA) according to the specification, CAL27 cells were transfected with si-E2F7#1, si-E2F7#2 and si-con, meanwhile, TCA-83 cells were transfected with pcDNA3.1-E2F7 and pcDNA3.1. After 48 h, the transfection efficiency was tested by qPCR.

### qRT-PCR

Total RNA of OSCC cells was extracted by applying a Trizol reagent following the instructions. Utilizing a High Capacity cDNA Reverse Transcription Kit (Applied Biosystems, UK), cDNA was synthesized. Then qPCR was executed on a ABI7300 real-time PCR machine (Helsinki, Finland) utilizing Power SYBRs Green PCR Master Mix (Applied Biosystems) by employing GAPDH as an endogenous control. The relative expression level was calculated utilizing the 2^-ΔΔCt^ method. Table 1 illustrated the primes utilized in qRT-PCR.

**Table 1:** The primers utilized in qRT-PCR

Name	Sequences
E2F7 forward	5′-TCTGAACCCGACTGTCCCTCTT-3’
E2F7 reverse	5′-TTTGGCAGCCACATCCAGAGTG-3’
GAPDH forward	5′-TGTGTCCGTCGTGGATCTGA-3’
GAPDH reverse	5′-CCTGCTTCACCACCTTCTTGA-3’

### Western blotting analysis

RIPA lysis buffer including protease inhibitor (Madison, WI, USA) was utilized to isolate total protein. The proteins were separated by 10% SDS-PAGE and transferred onto a PVDF membrane (Millipore, NY, USA). Subsequently, the membrane was blocked with 5% skim milk for 1 h. Then the membrane was incubated with primary antibodies overnight at 4 °C, and hatched with secondary antibodies for 1 h at room temperature. With an ECL Kit (Millipore, NY, USA), the proteins were visualized and tested utilizing an ImageJ software (Bio-Rad, Hercules, CA). Western blotting analysis was executed utilizing the primary antibodies: anti-E2F7 (1: 1000, ab245655, Abcam, MA, USA), E-cadherin (1:1000, #14472, Cell Signaling Technology, Inc.), N-cadherin (1:1000, #13116, CST), Vimentin (1:1000, #5741, CST), Snail (1:1000, #3879, CST), GAPDH (1:5000, #5174, CST) and the secondary antibodies (1:5000, #7076 or #7074, CST). The expression of GAPDH was a control.

### Cell proliferation and colony formation assays

After 48-h transfection, cell proliferation was carried out using Cell Counting Kit-8 kit (CCK-8, Dojindo, Tokyo, Japan). In brief, transfected cells (5000 cells/well) were added to 96-well plates. At 24, 48 and 72 h, 10 μl CCK8 solution was plated to each well and cultivated for another 1.5 h. The OD_450_ was assessed by a microplate reader.

For colony formation assay, transfected cells (1000 cells/well) were seed in 6-well plates and grown in RPMI-1640 serum medium for 14 days. Moreover, every 3 days, the medium was replaced. The colonies cells were fixed by utilizing 4% paraformaldehyde for 30 min and then were dyed with 0.1% crystal violet for 30 min. Lastly, the number of colonies was counted.

### Transwell invasion and migration assay

The invasion and migration of OSCC cells were evaluated by applying Transwell inserts pre-coated with or without Matrigel. Briefly, the transfected cells (1× 10^5^) in 100 μl of serum-free medium were seeded on the top chamber of 24-well plates; whilst 600 μl RPMI-1640 medium (supplying with 10% FBS) was added to the lower chamber. Incubating for 24 h, the cells were fixed with 4% polyoxymethylene for 30 min and dyed with 0.1% crystal violet for 10 min at 37 °C. Finally, a light microscope at magnification × 200 was utilized to visualize the outcomes. The number of cells was analyzed on ImageJ software (Bio-Rad, Hercules, CA). Each assay was executed in triplicate.

### Statistical analysis

All statistical data were analyzed with SPSS 22.0 software and GraphPad Prism 7.0 software. Comparisons between pairs groups were tested by Student’s t test. Comparisons among 3 or more than 3 groups were analyzed by One-way analysis of variance (ANOVA) followed by a Tukey’s post-hoc test. Data was presented as the Mean ± SD. *p* < 0.01 was regarded as statistical difference.

## Results

### E2F7 is up-regulated in OSCC tissues and associated with the prognosis of OSCC patients

**Fig. 1:**
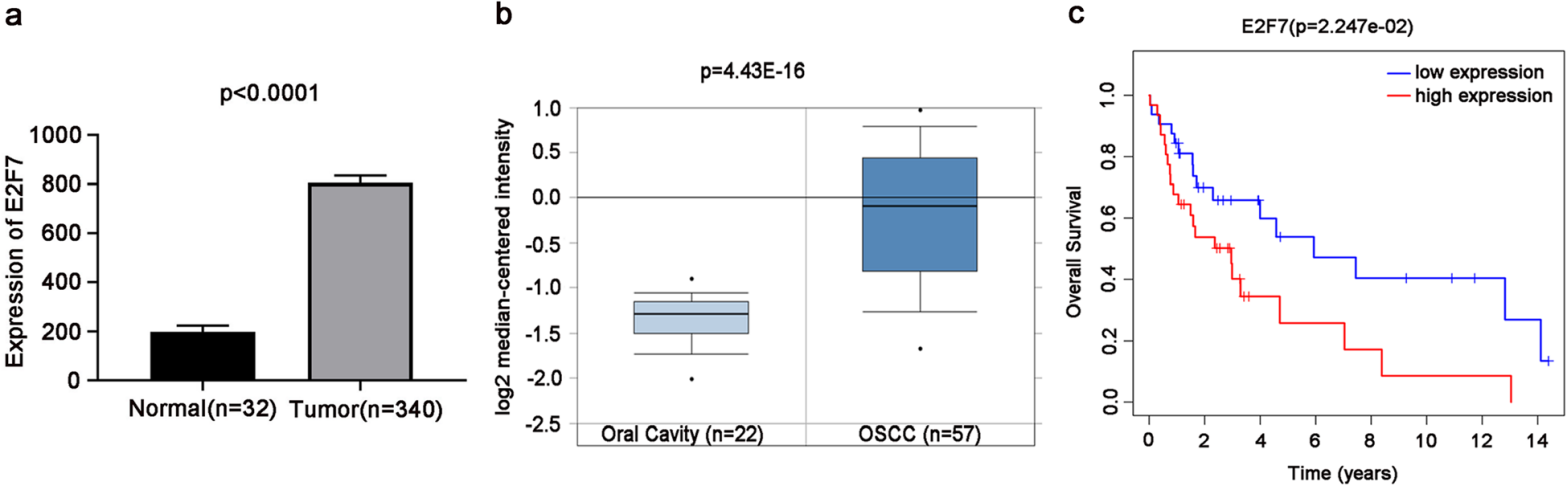
E2F7 is high-expressed in OSCC tissues and E2F7 high-expression predicts worse prognosis in patients with OSCC. **a** Basing on TCGA database, the expression level of E2F7 in OSCC tissues (*n* = 340) and normal samples (*n* = 32) were detected. **b** The expression level of E2F7 in OSCC tissues (*n* = 57) and oral normal samples (*n* = 22) were detected on the basis of ONCOMINE database. **c** The overall survival of E2F7 in OSCC patients was analyzed by Kaplan-Meier analysis

To examine whether E2F7 was correlated with OSCC development, we primarily analyzed E2F7 expression in OSCC tissues by utilizing RNA-Seq data from TCGA. We found that E2F7 expression was higher in the majority of OSCC tissues (*n* = 340) than in the non-tumor samples (*n* = 32) (Fig. 1a, *p* < 0.0001). Besides, E2F7 expression was higher in OSCC tissues (*n* = 57) than in normal tissues (*n* = 22) ( Fig. 1b, *p*= 4.43E-16) from ONCOMINE database. In addition, E2F7 high expression was concerned with a worse prognosis in patients with OSCC (Fig. 1c, *p* = 0.02247). These consequences demonstrated that E2F7 might be regarded as a prognostic factor for OSCC patients.

### Over-expression and deficiency of E2F7 in OSCC cells

**Fig. 2:**
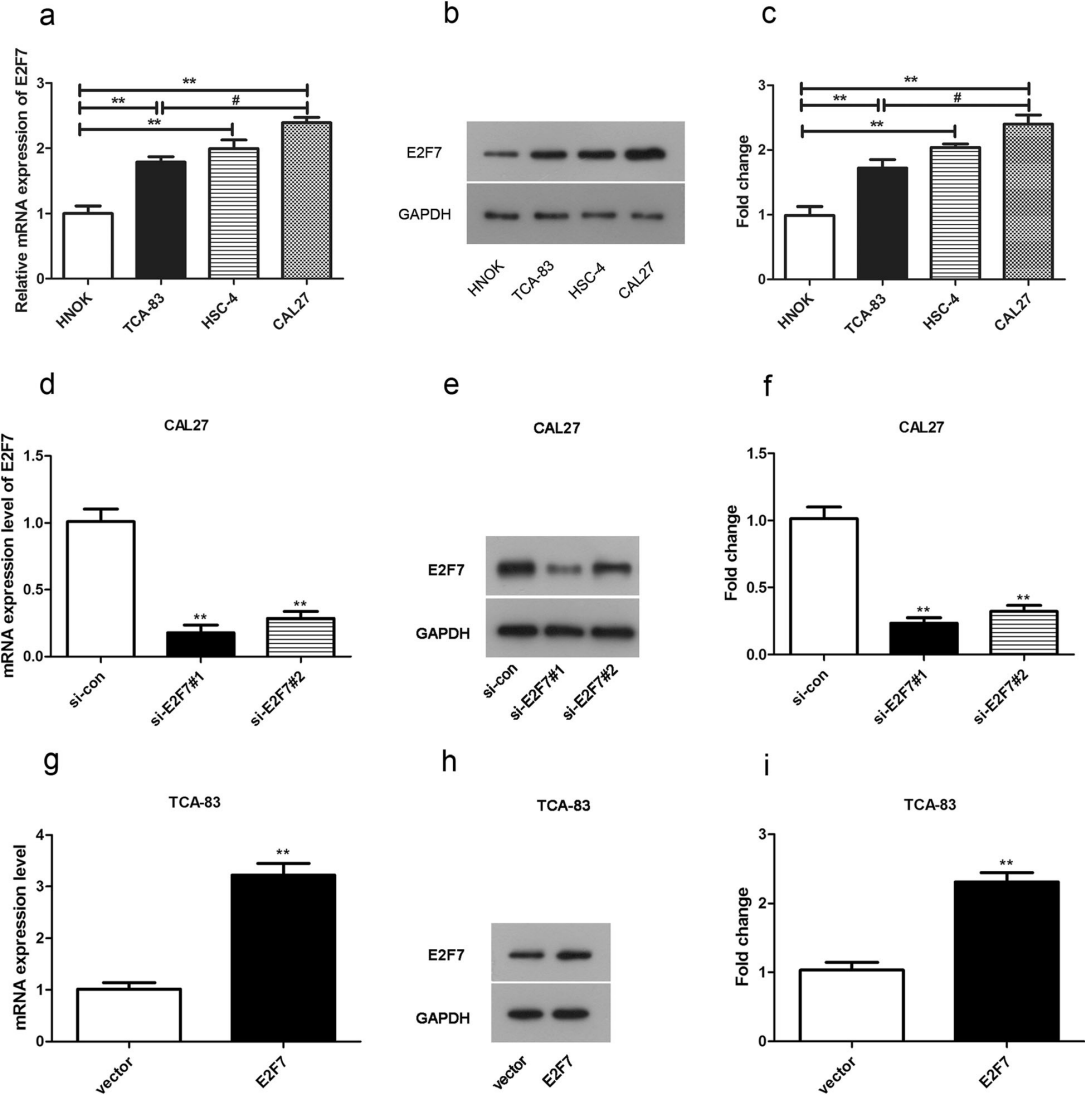
The levels of E2F7 in OSCC cell lines. **a**-**c** The mRNA and protein levels of E2F7 in HNOK, TCA-83, HSC-4, CAL27 and cell lines. **p < 0.01 vs. HNOK group. **d**-**f** The mRNA and protein expression of E2F7 in CAL27 cells. ** *p* < 0.01 vs. si-con group. **g**-**i** The mRNA and protein expression levels of E2F7 in TCA-83 cells. ** *p* < 0.01 vs. vector group

Furthermore, we explored the expression of E2F7 in OSCC cell lines. Firstly, we inquired the levels of E2F7 by using TCA-83, HSC-4, CAL27 3 different OSCC cell lines and a control cell line HNOK. Compared to HNOK cells, a visibly over-expression of E2F7 mRNA and protein expression was found in all tested OSCC cell lines (Fig. 2a-c), which was consistent with the outcomes of the databases analysis. Furthermore, E2F7 mRNA and protein expression levels were higher expressed in CAL27 cell line and lower expressed in TCA-83 cell line than other detected OSCC cell lines (Fig. 2a-c). Hence, detection of E2F7 knockdown effects were executed in CAL27 cell line and the impacts of E2F7 over-expression were tested in TCA-83 cell line in the following assays. As presented in Fig. 2d-f, si-E2F7#1 and si-E2F7#2 lessened the mRNA and protein expression of E2F7 in CAL27 cells. In addition, the knockdown efficiency of si-E2F7#1 was higher than si-E2F7#2, thus si-E2F7#1 was utilized in the subsequent experiments. Moreover, pcDNA3.1-E2F7 elevated the mRNA and protein levels of E2F7 compared to vector group in TCA-83 cells (Fig. 2g-i).

### Depletion of E2F7 represses cell growth of CAL27 cells whereas high-expression of E2F7 accelerates the growth of TCA-83 cells

**Fig. 3:**
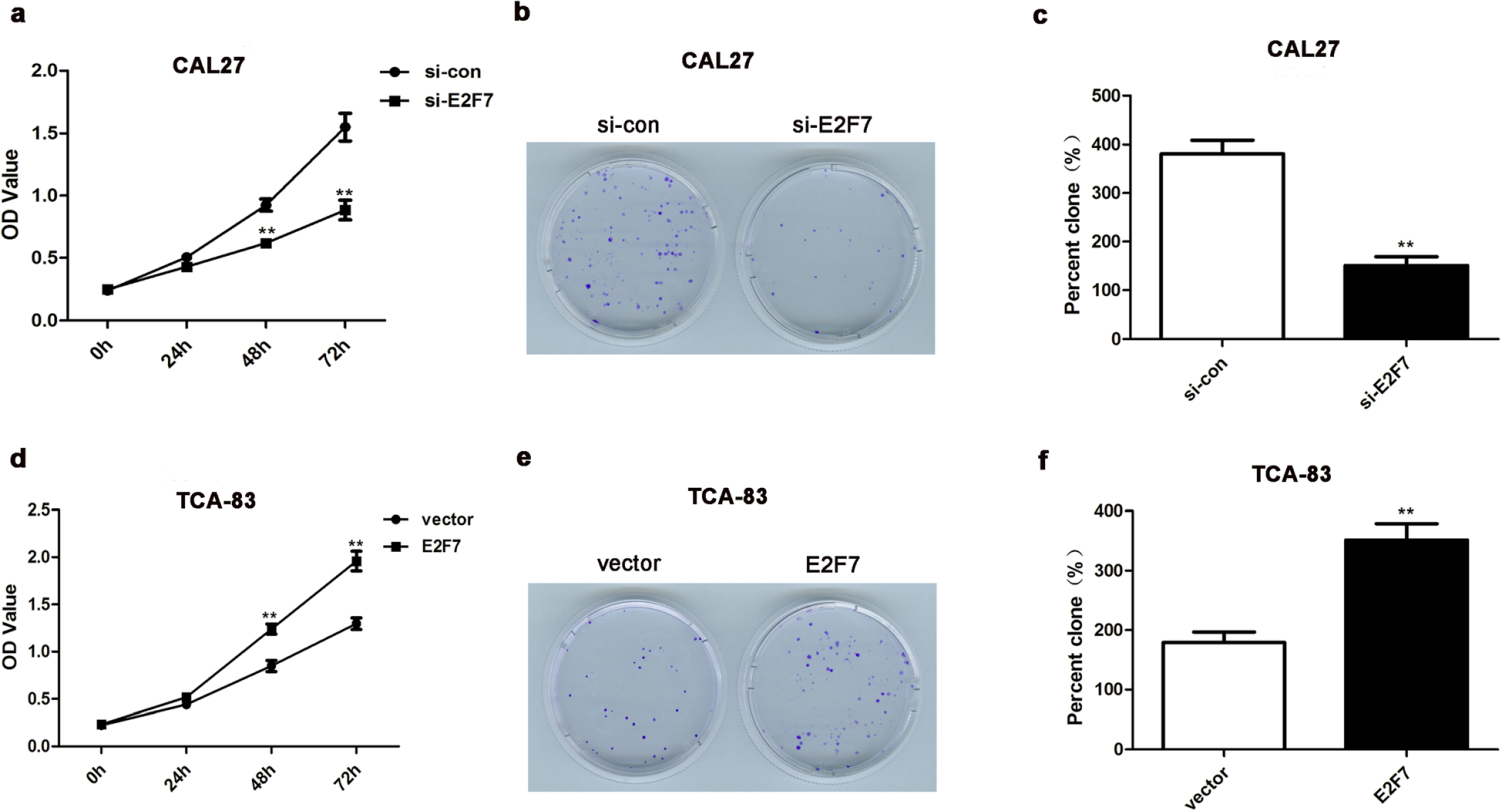
The influences of E2F7 on the growth of OSCC cells. **a** CCK8 assay displayed that E2F7 knockdown suppressed CAL27 cell proliferation. **p < 0.01 vs. si-con group. **b**-**c** Colony count statistics showed colony formation ability. **p < 0.01 vs. si-con group. **d** Over-expression of E2F7 accelerated TCA-83 cell proliferation, which was tested by CCK8. ** *p* < 0.01 vs. vector group. **e** Representative images of colonies formed by TCA-83 cells. **f** Quantification of (**e**). ** *p* < 0.01 vs. vector group

To determine the influences of E2F7 on OSCC cell growth, we executed CCK8 and colony formation assays. Compared with si-con group, the OD_450_ value was reduced in si-E2F7 group (Fig. 3a), proving that E2F7 deficiency decreased CAL27 cell proliferation, as revealed by CCK8 assay. Moreover, after cultivated for 48 h and 72 h, E2F7 ablation significantly reduced CAL27 cell proliferation, however, no significant impact was displayed at 24 h (Fig. 3a). As shown in Fig. 3b-c, E2F7 silencing repressed the colony formation abilities of CAL27 cells. Over-expression of E2F7 facilitated TCA-83 cell proliferation after cultivated for 48 h and 72 h, yet no significant influence at 24 h was displayed (Fig. 3d). Moreover, over-expression of E2F7 elevated the colony formation abilities of TCA-83 cells (Fig. 3e-f). These findings indicated that E2F7 depletion had a suppressive impact and E2F7 over-expression had a promotive effect on the growth of OSCC cells.

### The impacts of E2F7 on the invasion and migration of OSCC cells

**Fig. 4:**
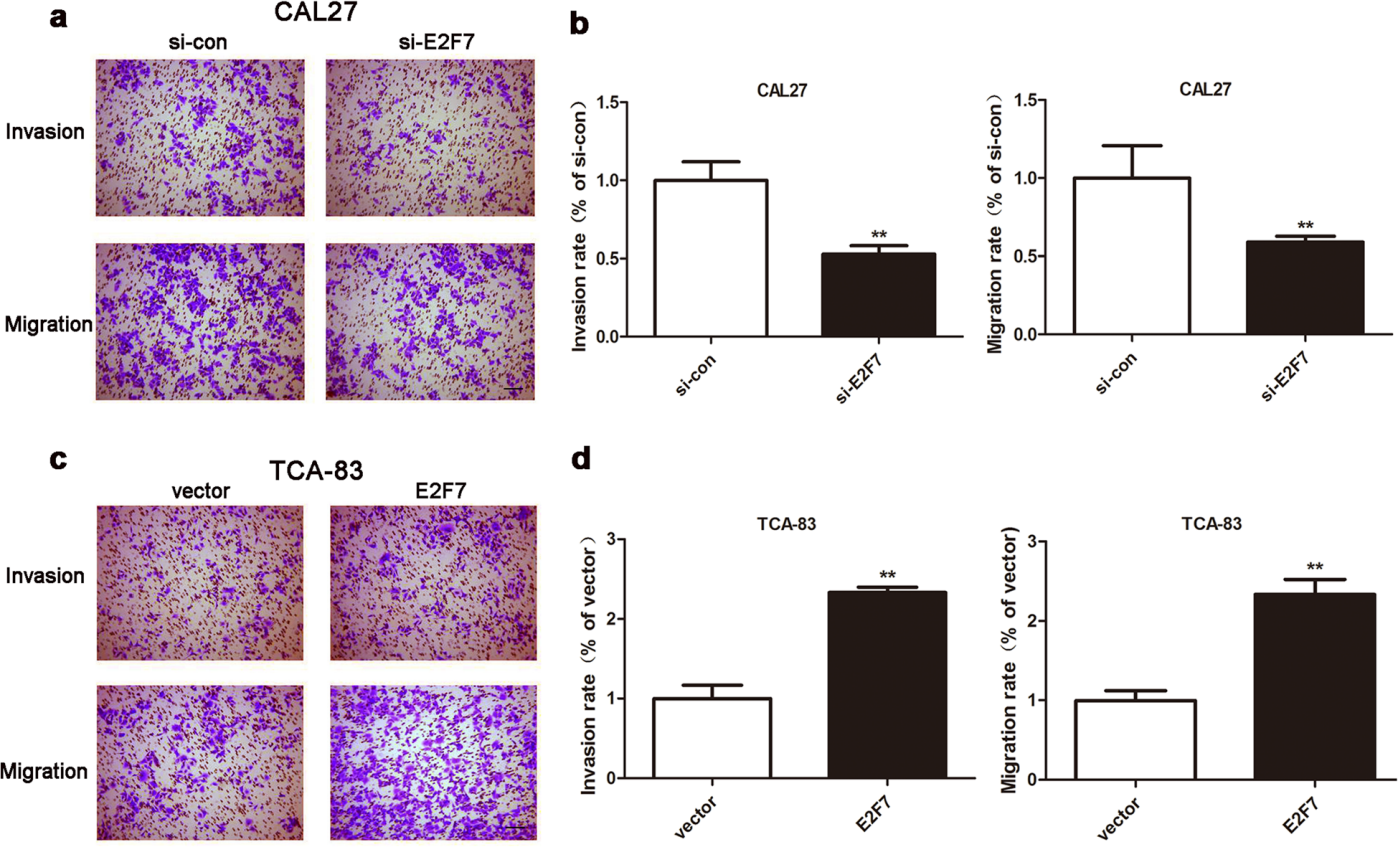
Transwell assay was used to detect the impacts of E2F7 on OSCC cell migration and invasion. **a**-**b** The invasive and migrated abilities of E2F7 depleted CAL27 cells. Scale bar: 200 μm. **p < 0.01 vs. si-control group. **c**-**d** The invasive and migrated abilities of E2F7 high-expressed TCA-83 cells. Scale bar: 200 μm. **p < 0.01 vs. vector group

To further explore the functions of E2F7 on OSCC cell invasion and migration, the transwell assay was used. Ablation of E2F7 distinctly decreased the number of invasive and migrated CAL27 cells (Fig. 4a-b). Inversely, high-expression of E2F7 increased the number of invasive and migrated TCA-83 cells (Fig. 4c-d). In summary, the above data ulteriorly indicated that E2F7 might act a promoting effect on OSCC cell invasion and migration.

### E2F7 can regulate EMT in CAL27 and TCA-83 cells

**Fig. 5:**
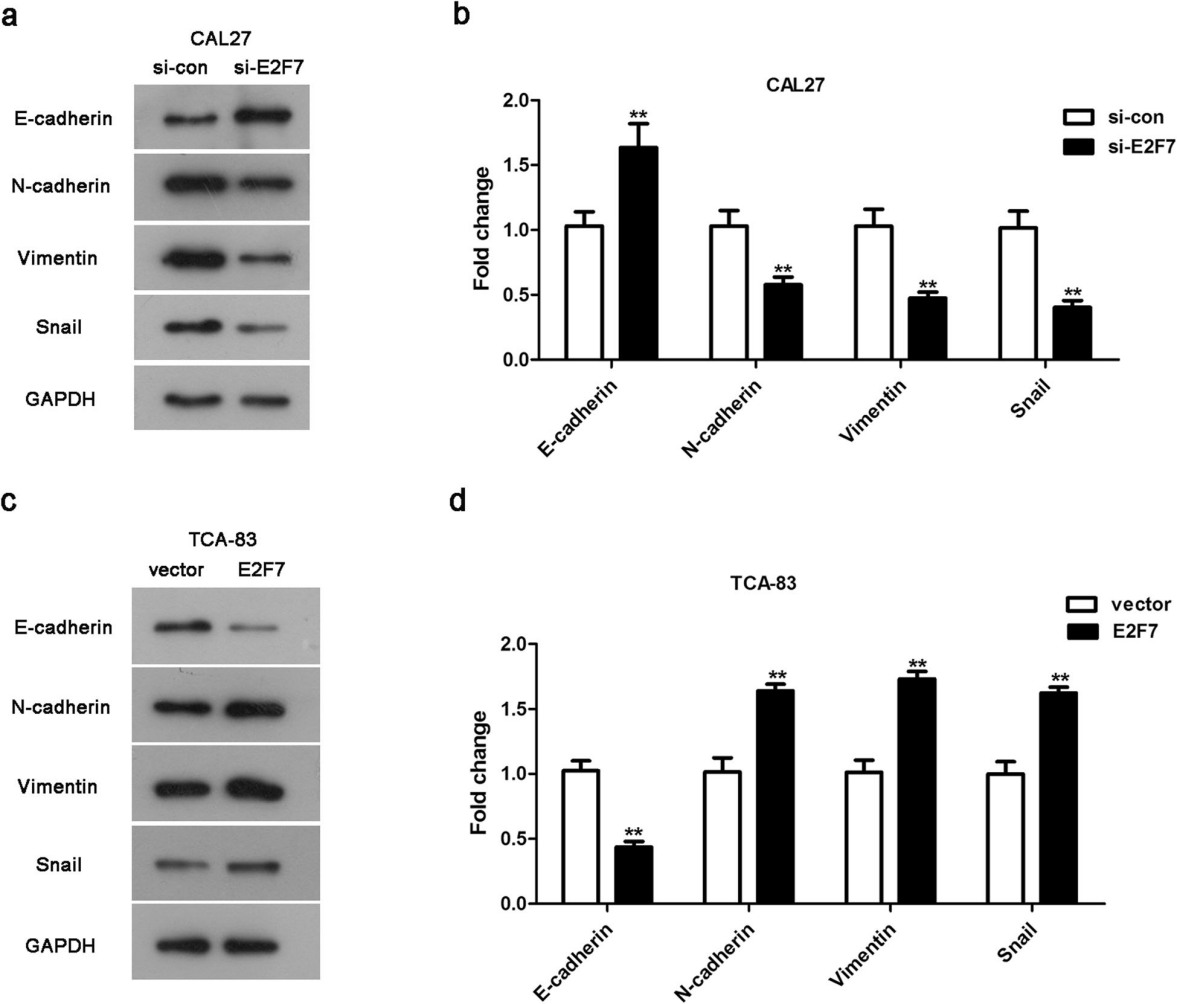
The impacts of E2F7 on OSCC cells might be modulated by EMT. **a**-**b** Through western blotting, the protein levels of E-cadherin, Ncadherin, Vimentin and Snail in CAL27 cells were detected. ** *p* < 0.01 vs. si-con group. **c**-**d** The protein levels of E-cadherin, Ncadherin, Vimentin and Snail in TCA-83 cells were tested by western blotting. **p < 0.01 vs. vector group

To gain a deeper comprehending on the mechanisms by which E2F7 affects OSCC cell growth and motility, the protein levels of E-cadherin, N-cadherin, Vimentin and Snail were tested by western blotting. As presented in Fig. 5a-b, E2F7 ablation enhanced the level of E-cadherin, whilst knockdown of E2F7 significantly receded N-cadherin, Vimentin and Snail levels in CAL27 cells. Additionally, E2F7 over-expression reduced the level of E-cadherin in TCA-83 cells (Fig. 5c-d). Whereas, N-cadherin, Vimentin and Snail levels were heightened by high-expression of E2F7 in TCA-83 cells (Fig. 5c-d). All the above findings hinted that E2F7 regulated OSCC cell motility partially via EMT.

## Discussion

Some transcription factors have been authenticated as key regulators of a variety of cellular processes, particularly for cancer initiation and progression [15, 17]. As a pro-tumorigenic transcriptional factor, although elevated E2F7 expression was found in gliomas [13], the impacts of E2F7 in OSCC are still not fully investigated in depth. In this study, we found that E2F7 was over-expressed in OSCC tissues and high expression of E2F7 was correlated with a worse prognosis in OSCC patients. Moreover, E2F7 depletion inhibited the growth, invasion and migration of CAL27 cells, whilst E2F7 high-expression exerted the opposite influences on TCA-83 cells, which were all possible realized through modulating EMT. Hence, our consequences showed that E2F7 might considered as a helpful marker for the treatment of OSCC.

The E2F transcription factor family is vital for the regulation of cell growth, differentiation, apoptosis, DNA damage responses and so on [7]. Due to functional and structural feature, E2Fs are split into activating agent (E2F1, E2F2 and E2F3a) and inhibitors (E2F3b, E2F4–8) [14]. More notably, the main effects of E2F1 is to be utilized as checkpoints for cell proliferation and apoptosis [18]. Recently, it has indicated that E2F7 is considered to be a major regulator of E2F1 activity [13, 14]. As a relatively novel transcription factor, E2F7 was revealed to modulate cell cycle by suppressing G1-S genes expression in late S phase [19, 20]. E2F1 and E2F7 can form a heterodimer and recruit a co-repressor C-terminal binding protein (CtBP) to inhibit G1-S transcription [21, 22]. One of the most important hallmarks for cancers is uncontrolled cell growth, and mutations in tumor cells are usually obtained in genes that firsthand modulate their cell cycle [23, 24]. A new discovery demonstrated that knockdown of E2F7 repressed endometrial cancer cell growth [25]. Intriguingly, E2F7 was indicated to highly expressed in gliomas tissues [13]. Consistent with the results in endometrial cancer and glioma, we discovered that E2F7 executed roles in facilitating OSCC cell growth and invasion, together indicating that E2F7 is an oncogene.

Moreover, our study also found that E2F7 regulated OSCC cell motility partially through EMT. EMT is an important process in tumors that affects the key process by transforming epithelial cells into morphogenesis steps of cells with mesenchymal properties [26]. Induction of EMT is critical for OSCC cancer metastasis, concerning several phenotypic change of tumor cells which are modulated by many EMT markers [27, 28]. When EMT occurs, E-cadherin (a vital cell-to-cell adhesion molecule) expression is down-regulated, whereas the expression of N-cadherin (associate with a cadherin switching process), Vimentin (a pivotal impact in cell migration) and Snail (an EMT-related transcription factor) are up-regulated [26, 29, 30]. As reported, co-assessment of E-cadherin and Vimentin might be a worthy tool for forecasting outcomes of OSCC patients [31]. Ozaki-Honda et al. [32] exhibited that N-cadherin was of great value on prognosis prediction in OSCC patients. It has found that Snail was up-regulated in oral cancer cells [33]. In gallbladder carcinoma, E-cadherin and vimentin protein levels were affected by miR-30a-5p depletion, and these influences were partly weakened by E2F7 suppression [34]. In LUAD cells, SNHG6 facilitated cell migration and the activity of EMT by targeting miR-26a-5p/E2F7 axis [12]. Collectively, in line with these findings, our consequences indicated that the effects of E2F7 on the growth, invasion and migration of OSCC cell were potentially modulated through EMT.

Taken together, we concluded that E2F7 was highly expressed in OSCC tissues and cell lines firstly. Besides, E2F7 high-expression was concerned with poor overall survival in OSCC patients for the first time. Moreover, the facilitating influences of E2F7 on the growth, invasion and migration of OSCC cells might be regulated via EMT. Our findings will offer new insights in the modulation of E2F7 on OSCC pathogenesis. However, there are some disadvantages in our study. First, there may be more mechanisms or pathways involved in the influences of E2F7 on OSCC. Second, the in-vivo studies could be need to ulteriorly confirm our consequences. We will report these results in future articles.

## Data Availability

The data and material in this study is available from the corresponding author on reasonable request.
